# Introduction to the *RSC Advances* themed collection on the photoluminescence of lanthanide-doped phosphor materials

**DOI:** 10.1039/d5ra90094h

**Published:** 2025-09-23

**Authors:** Ram Sagar Yadav, Bryce S. Richards, Joanna Pisarska, Xinyu Ye

**Affiliations:** a Institute of Science, Banaras Hindu University Varanasi 221 005 India ramsagaryadav@gmail.com; b Institute of Microstructure Technology, Karlsruhe Institute of Technology Hermann-von-Helmholtz-Platz 1, 76344 Eggenstein-Leopoldshafen Germany; c Institute of Chemistry, University of Silesia Szkolna 9 Street Katowice 40-007 Poland; d Faculty of Materials, Metallurgy and Chemistry, Jiangxi University of Science and Technology Ganzhou 341000 P. R. China

## Abstract

Ram Sagar Yadav and his team introduces the *RSC Advances* themed collection on photoluminescence of lanthanide-doped phosphor materials.
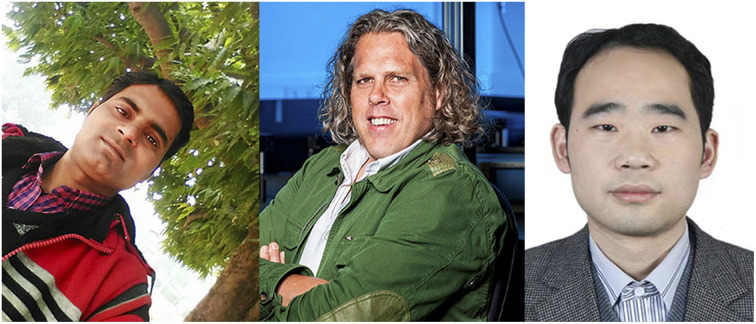

Laser spectroscopy is an essential tool to study the optical properties of the lanthanide-based materials. Lanthanide ions are most stable in a trivalent ion (Ln^3+^) state, and produce multicolor photoluminescence due to the presence of various distinct energy levels in which some are meta-stable. Each energy level can be excited with a certain excitation wavelength, with the emitted light of lanthanide ions covering emissions in the ultraviolet, visible and near infrared regions. The lanthanide ions also enable upconversion (UC) and downshifting (DS) of the incident photons. The low energy incident photons are converted into a high energy photon in the UC process while the conversion of a high energy photon into a low energy photon is termed as luminescent down-shifting (LDS). The UC process is helpful for measuring the temperature sensing sensitivity of different phosphor materials, as it depends on the fluorescence intensity ratio (FIR) of the two thermally coupled (TC)/non-thermally coupled (NTC) levels. In these cases, the emission intensity of one level is found to increase while that of the other level decreases when the external temperature of the sample is increased, and *vice versa*. The plot between FIR-based sensitivity and external temperature gives the temperature sensing, which can be examined in other lanthanide-doped phosphor materials. Due to various properties of lanthanide ions, lanthanide-doped phosphor materials can be utilised for different technological applications in various fields, such as display devices, white light emitting diodes (w-LEDs), solar cells, anti-counterfeiting, information storage, optical heating, temperature sensing, and bio-imaging.^1-8^

This themed collection considers the synthesis of lanthanide-doped phosphor materials, and provides insight into the structural and optical properties of these materials, including phosphors, nano-phosphors and phosphor composites.

The development of lanthanide luminescence has been summarized in terms of their present, past and future advancements including the practical applications of different lanthanide doped photoluminescent materials in various technological fields (https://doi.org/10.1039/D3RA00991B). In one contribution to this series, the photoluminescence activities of rare earth self-activated phosphors are discussed. This activity occurs through energy transfer between the rare earth ions and niobate/vanadate host materials *via* different processes, such as UC, DS, quantum cutting (QC), *etc.* Their applications in different fields are also discussed (https://doi.org/10.1039/D3RA00629H).

Another contribution to the series focuses on the combination of lanthanide (Eu^3+^) and transition metal (Mn^4+^) co-doped LaAlO_3_ phosphor, enabling energy transfer between them, which can be used for horticultural lighting purposes (https://doi.org/10.1039/D3RA03241H). The role of Fe^2+^ ions on Er^3+^ doped nanoparticles was also investigated for the use as a sensing material (https://doi.org/10.1039/D3RA04645A), and the sonochemically-assisted synthesis of a silver-supported α-Fe_2_O_3_ (SA@Ag@IONC) nanocomposite is highlighted as a better choice to breakdown hazardous dye in wastewater treatment (https://doi.org/10.1039/D3RA03315E). NaLi_2_PO_4_:Cu(ii) and NaLi_2_PO_4_:Cu(i) materials are highlighted as good candidates for radiation dosimetry with a wide dose response (https://doi.org/10.1039/D3RA02498A), and the synthesis of ZrTiO_4_ nanorods by a solution combustion method is reported alongside the photoluminescence, antibacterial, X-ray/gamma ray absorption, supercapacitor and sensor applications of the nanorods (https://doi.org/10.1039/D3RA00908D).

Lanthanide doped phosphor materials have been widely investigated by different groups of researchers in this collection. Initially, the DS photoluminescence of Eu^2+^, Ce^3+^, Eu^3+^, Tb^3+^, Dy^3+^, Ho^3+^, Tm^3+^ and Pr^3+^ doped phosphor materials have been investigated in detail. The structure and morphology of the phosphor materials were modified *via* the incorporation of alkali as well as alkaline earth metal ions. These properties were also modified through the incorporation of transition metal ions. These processes are highly favourable for improving the photoluminescence intensity of the phosphor materials. The photoluminescence intensity of a Eu^3+^ doped ZnGa_2_O_4_ phosphor was improved significantly *via* doping with Ca^2+^ and Mg^2+^ ions, which leads to the production of bright tunable photoluminescence (https://doi.org/10.1039/D3RA03215A). Energy transfer has been reported in the Dy^3+^/Eu^3+^ co-doped Na_4_Ca_4_Si_6_O_18_ phosphors in which the energy transfer takes place from Dy^3+^ to Eu^3+^ ions, which also produces colour-tunable photoluminescence (https://doi.org/10.1039/D3RA03229A). Energy transfer has also been studied in the Tm^3+^/Tb^3+^/Eu^3+^ co-doped Sr_4_Nb_2_O_9_ phosphors, which leads to the production of colour tunable luminescence due to energy transfer from Tm^3+^ to Tb^3+^ and Eu^3+^ ions. Lifetime analysis also supports an efficient energy transfer from Tm^3+^ to Tb^3+^ and Eu^3+^ ions (https://doi.org/10.1039/D3RA05519A).

Finally, researchers have studied the UC process in different lanthanide doped phosphor materials in detail. The UC process has been studied in Tm^3+^; Yb^3+^ co-doped Zn_2_TiO_4_ phosphors using a solid-state reaction route. The phosphor produces an intense blue colour due to ^1^G_4_ → ^3^H_6_ transition under 980 nm excitation, which finds its applications in anti-counterfeiting fields (https://doi.org/10.1039/D3RA03238H). Enhancement in the photoacoustic and green UC emissions of the Er^3+^/Yb^3+^ co-doped Gd_2_O_3_ phosphor has been achieved using Mg^2+^/Zn^2+^ ions under 980 nm excitation (https://doi.org/10.1039/D3RA03041E). This phosphor is also useful for the detection of fingerprints and security applications. The Er^3+^/Yb^3+^ co-doped NaZr_2_(PO_4_)_3_ material gives intense green UC with a good FIR under 980 nm excitation, which is useful for temperature sensing applications (https://doi.org/10.1039/D3RA02126B).

We thank all colleagues who participated in this special collection for their enlightening scientific contributions.

## Conflicts of interest

There are no conflicts of interest to declare.
